# Implementation and evaluation of a nurse-led intervention to augment an existing residential aged care facility outreach service with a visual telehealth consultation: stepped-wedge cluster randomised controlled trial

**DOI:** 10.1186/s12913-023-10384-z

**Published:** 2023-12-18

**Authors:** Carla Sunner, Michelle Giles, Jean Ball, Roslyn Barker, Carolyn Hullick, Christopher Oldmeadow, Maralyn Foureur

**Affiliations:** 1 Hunter New England Local Health District, Newcastle, 2300 Australia; 2 University of Newcastle, Callaghan, 2308 Australia; 3 Hunter Medical Research Institute, New Lambton, 2305 Australia

**Keywords:** Telehealth, Nursing home, Hospital transfers, Patient safety, Emergency care, Older people

## Abstract

**Background:**

Up to 75% of residents from residential aged care facilities (RACF) are transferred to emergency departments (ED) annually to access assessment and care for unplanned or acute health events. Emergency department presentations of RACF residents can be both expensive and risky, and many are unnecessary and preventable. Processes or triage systems to assess residents with a health event, prior to transfer, may reduce unnecessary ED transfer. The Aged Care Emergency (ACE) service is a nurse-led ED outreach service that provides telephone support to RACF nurses regarding residents’ health events. This service is available Monday to Friday, 8am to 4 pm (ED ACE hours). The primary objective of this study was to assess whether the augmentation of the phone-based ED ACE service with the addition of a visual telehealth consultation (VTC) would reduce RACF rate of ED presentations compared to usual care. The secondary objectives were to 1) monitor presentations to ED within 48 h post VTC to detect any adverse events and 2) measure RACF staff perceptions of VTC useability and acceptability.

**Methods:**

This implementation study used a stepped wedge cluster randomised controlled trial design. Study settings were four public hospital EDs and 16 RACFs in two Local Health Districts. Each ED was linked to 4 RACFs with approximately 350 RACF beds, totalling 1435 beds across 16 participating RACFs. Facilities were randomised into eight clusters with each cluster comprising one ED and two RACFs.

**Results:**

A negative binomial regression demonstrated a 29% post-implementation reduction in the rate of ED presentations (per 100 RACF beds), within ED ACE hours (IRR [95% CI]: 0.71 [0.46, 1. 09]; *p* = 0.122). A 29% reduction, whilst not statistically significant, is still clinically important and impactful for residents and EDs. A post-hoc logistic regression demonstrated a statistically significant 69% reduction in the probability that an episode of care resulted in an ED presentation within ED ACE hours post-implementation compared to pre-implementation (OR [95% CI]: 0.31 [0.11, 0.87]; *p* = 0.025).

**Conclusion:**

Findings have shown the positive impact of augmenting ACE with a VTC. Any reduction of resident presentations to a busy ED is beneficial to healthcare overall, but more so to the individual older person who can recover safely and comfortably in their own RACF.

**Trial registration:**

Australian New Zealand Clinical Trials Registry (ID ACTR N12619001692123) (02/12/2019) https://www.anzctr.org.au/Trial/Registration/TrialReview.aspx?id=378629andisReview=true

**Supplementary Information:**

The online version contains supplementary material available at 10.1186/s12913-023-10384-z.

## Background

While unnecessary ED presentations are a major issue in all hospital EDs, the risk is more so for the vulnerable RACF resident. The ED environment can be distressing and disorientating for an older person and can expose the RACF resident to unnecessary risks such as physical harm, psychological distress, and iatrogenic complications such as delirium, falls, medication errors, pressure injuries and even death [1, 2]. Furthermore, RACF residents have a very high rate of ambulance use, up to four-times higher than that of their community counterparts [1] with the cost of a single transfer by ambulance of a resident from an RACF to an ED in one Australian study reported as over $1800 [3]. These issues highlight an urgent need to provide improved primary care services for RACF nurses to support and manage residents whilst remaining in the RACF to avoid unnecessary presentations to ED where possible.

In the last decade, an extraordinary increase in people aged over 65 years has been observed in almost all countries [4]. In Australia, from 2010 to 2020, the population aged over 85 years increased by 110% [5]. Consequently, the number of people needing supportive care in residential aged care facilities (RACF) between 2010 and 2020 has increased by 13% and the number of women almost double the number of men [6]. In 2022 there were 178 000 people living in RACFs in Australia [6] of these residents 96.1% were known to have physical or cognitive impairment [5] that may cause some sort of limitation, restriction, or impairment to their activities of daily living.

More and more older adults are spending a longer time living independently at home before transitioning to a RACF. This transition often occurs when individuals are in poorer health and have more complex care needs. The chronic and intricate physical and cognitive challenges faced by residents in residential aged care facilities (RACF) often involves coexisting conditions like frailty and cognitive impairment. These conditions render them more vulnerable, especially when it comes to advocating for their preferred treatment goals [7].

RACFs face numerous challenges in managing residents with increased healthcare demands, resulting in greater workloads for staff and a lack of timely access to primary healthcare doctors and geriatric care expertise [8]. The shortage and unavailability of primary healthcare providers or general practitioners (GPs) can be attributed to various reasons, including complex issues in residents with greater health needs, poor renumeration for the GPs’ time, and conflicting practice commitments [9]. Starting in July 2023, Australian RACF providers must have an RN on-site 24/7 at each facility [10]. However, when RACF nurses can't access timely GP advice, it still results in unnecessary resident transfers to the ED.

Establishing goals of care are particularly important for older residents as they outline what a resident (or the carer) wants to achieve during an episode of care, within the context of their medical situation [11]. When these goals are not well articulated and established, residents may receive complex and invasive investigations, treatments, and procedures in ED, that are not always beneficial.

The reliance on others to make decisions about their health care needs has contributed to higher RACF resident presentations to hospital emergency departments (ED), compared with their community counterparts [12]. Of major concern is the finding that RACF residents are often discharged from an ED presentation without any need for treatment or an ongoing management plan. Emergency Department presentations, without subsequent admission to hospital, have been reported to be up to 40% of all RACF resident presentations to ED [3, 12].

Other RACF outreach programs like INTERACT [13], admission avoidance [14], and INTERCARE [15] offer care pathways and guidance to RACF nurses for RACF residents and hospital avoidance. However, only one other study [16] was a nurse-led, included visual telehealth consultation, and was situated in the emergency department of a hospital similar to the study presented in this paper.

A model of care that aims to improve primary care services for the older person in RACFs is the Aged Care Emergency (ACE) service. The Aged Care Emergency service provides outreach support to assist RACF nurses with decision-making and establishing clearly defined goals of care for the RACF resident, prior to a journey to ED [17]. The Aged Care Emergency service supports RACF nurses when the Primary health provider or general practitioner (GP) is unavailable and communicates care goals for resident transfers to the ED but does not replace the GP as the primary healthcare provider for RACF residents.

In its current form telephone calls to the ACE service are usually answered by Aged Care Service Emergency Team (ASET) registered nurses working in the ED who have advance practice knowledge in older person care. In consultation with the RACF nurses, ASET nurses provide support over the telephone, guided by evidence-based algorithms, to help determine whether transfer of the resident to ED is necessary [18]. Planning alternative pathways for residents with the use of the ACE service also means the RACF nurses, residents and carers not only have an opportunity to avoid an ED presentation but there are also further savings through avoiding the cost of ambulance transfer [19].

The ACE service is available 24 h a day, however it has two providers; the ED ACE service is available Monday to Friday between 8am and 4 pm (ED ACE hours) and a non-ED ACE service is provided by a separate primary health care provider for after-hours ACE calls.

A limitation of the ACE service is that the calls are by telephone only. More recently the emergence of visual telehealth has been utilised in health care. A recent scoping review found that the addition of Visual Telehealth Consultations (VTC) reduced hospitalisation of RACF residents [20] with one randomised controlled trial reporting that 71% of residents were successfully managed in the RACF with the use of visual telehealth [21]. However, reviews and meta-analyses of studies on the use of telehealth in RACFs highlight many poor-quality studies, with inconsistent outcome measures demonstrating a need for more robust and large-scale implementation studies [22]. We propose that a nurse-led ED ACE model, augmented by the addition of VTC and structured and documented communication processes, implemented within RACFs, has the potential to optimise the benefits of both ACE and VTC, to reduce avoidable RACF resident presentations to ED.

Findings presented in this paper arise from the Partnerships in Aged Care Emergency using Interactive Telehealth (PACE-IT) study [23], which had the following aims:

- to assess whether the augmentation of Age Care Emergency services through the addition of interactive Visual Telehealth Consultation (= Emergency Department Age Care Emergency Age Care Emergency/ Visual Telehealth Consultation VTC) for clinical decision-making, plus a structured and documented communication framework and telephone follow-up, reduced RACF resident presentations to ED compared to usual care within ED Age Care Emergency hours, 8am-4 pm Mon-Fri;
- to identify potential adverse events for residents who did not attend the ED, by conducting a 24-h follow-up survey and assessing RACF resident ED presentations within 48 h of a Visual Telehealth Consultation;
- to ascertain RACF staff perceptions of Visual Telehealth Consultation acceptability and useability within 48 h of participating in a Visual Telehealth Consultation;
- to assess uptake of Age Care Emergency / Visual Telehealth Consultation during ED Age Care Emergency hours.

Several secondary outcomes have already been reported, encompassing staff perception of barriers and enablers to implementation and sustainability three-months post-implementation, staff acceptability and engagement at the same time-point, as well as resident and family experiences with the intervention one-month post-implementation [24]. A separate report will present the cost consequence analysis.

The presented findings adhere to the Standards for Reporting Implementation Studies (StaRI) checklist [25] and comply with guidelines for reporting stepped wedge cluster randomised controlled trials, as outlined in the extension of the CONSORT 2010 statement [26]. The significance of the proposed intervention is its potential impact on improving care for older people that reside in RACFs and reducing the associated risks for residents with ED presentation and hospital admission.

## Methods

### Study design

This large scale implementation study used a stepped wedge cluster randomised controlled trial (RCT) design, chosen for its pragmatic approach allowing for rigorous evaluation in a real life setting [27]. The study protocol has been described previously [23]. The trial is registered with the Australian New Zealand Clinical Trials Registry (Trial ID ACTR N12619001692123).

### Setting

**Fig. 1:**
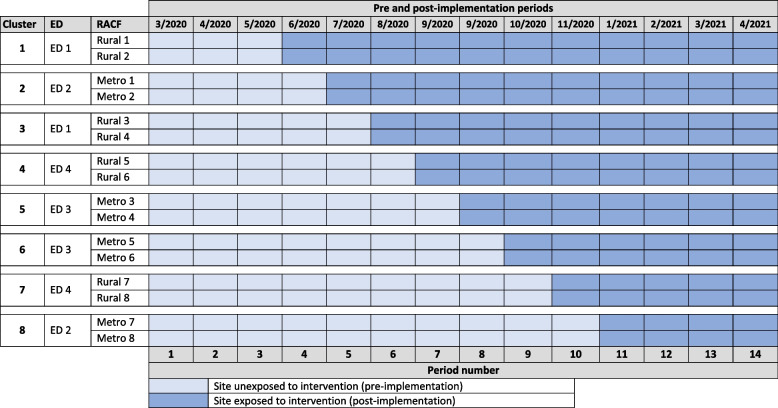
Data collection periods in PACE-IT stepped wedge design (Metro = metropolitan)

The study was conducted in four public hospital EDs in two Local Health Districts (LHDs) and 16 RACFS, in New South Wales (NSW), Australia. The EDs were selected for their metropolitan and rural locations; LHD A has two metropolitan and one rural ED and LHD B has one rural ED. The Australian Department of Health and Aged Care uses the Modified Monash Model (MMM) [28] to categorise different geographical locations based on Australian bureau of statistics. In this context, the participating EDs for LHD A had two EDs in a metropolitan city (MMM 1), and another is a large rural town (MMM 3) and LHD B primarily covers a large rural town (MMM 3). Each selected ED was linked with 4 RACFs with approximately 350 RACF beds, totalling 1435 beds across all 16 participating RACFs. For implementation purposes, facilities were organised into eight clusters for randomisation, by a statistician using a computer-generated randomisation sequence, with each cluster comprising one ED and two RACFs (Fig. 1); each ED was included in two clusters. The intervention was sequentially implemented in the RACFs in each cluster following an order determined by the randomisation process – the study “steps”—with a time interval of three to seven weeks between each step. Data were collected on episodes of care from 3 months prior to the commencement of the implementation of the intervention to 3 months after the date of the last intervention, resulting in 14 months of data for analysis.

An episode of care included all instances where an ED medical record was created for an RACF resident when:

- the resident was transferred to the ED, with or without an ED ACE call/VTC, or
- an ED ACE/VTC was attended, and the resident was not transferred to the ED.

NB: Individual residents may have had more than one episode of care within the study period.

For descriptive statistics, primary and secondary outcomes, as well as the post-hoc analysis, only episodes of care which occurred within ED ACE hours (Monday to Friday, 8am to 4 pm) were included. An extra analysis was conducted for comparison, including all episodes of care (non-ED ACE and ED ACE combined).

### Sample size

The feasibility study conducted in 2018 (not published) identified 82 RACF ED presentations/100 beds annually. With 16 RACFs contributing 1435 beds (‾X = 87) to this study, adopting the intervention in the sequence shown in Fig. 1, we estimated this design would have 80% power to detect a 35% relative reduction in ED presentations /100 beds annually (at 5% significance), assuming an intra-class correlation of 0.01.

### Participating sites

After the Executive Directors of LHD A and LHD B agreed to participate in the study, an expression of interest was emailed to RACF managers from both LHDs inviting them to participate. To be considered for inclusion in the PACE-IT project, RACFs in the two LHDs were eligible if they reported more than 40 RACF resident presentations to ED in 12 months. As well as the number of presentations, RACFs were required to have functioning Wi-Fi, internet capacity and willingness to commit a minimum of six staff for PACE-IT training. A letter of agreement was signed by the management of each RACF committing to the project for its duration. Individual consent was not required for RACF residents’ participation in this project as care provision was an extension of an existing service (ACE) and considered part of usual care for the resident in the event of an acute health episode.

### Intervention

The PACE-IT project augmented the pre-existing nurse-led ED ACE Model of Care (MOC) (Supplementary File 1- The essential elements of ACE [29]with the addition of VTC in LHD A. ACE consists of telephone calls from RACFs for consultation and is provided 24 h a day, by two separate services; the ASET nurse in the ED from 8am-4 pm and after hours by a separate primary health care provider. We refer to ED ACE, where an ASET nurse takes ACE calls within rostered hours: Monday to Friday, 8 am to 4 pm and non-ED ACE, where a separate health care provider takes after-hours ACE calls. However, LHD B had no pre-existing ACE service prior to the PACE-IT implementation, so ACE and PACE-IT were implemented simultaneously. LHD B had a budgeted project coordinator employed specifically to assist with the implementation process and data collection and additional Aged Care Emergency education and resources provided. The PACE-IT intervention provided an interactive ED ACE/VTC to enhance assessment using the ISBAR communication tool [30] (Supplementary File 2) and decision-making, with a 24 h follow up phone call for residents who did not present to ED. Refer to the ACE and VTC flowchart (Supplementary File 3) for further explanation.

Within 24 h of a VTC, where the resident did not attend the ED, a follow up phone call was conducted to monitor potential adverse events. Follow-up callers asked RACF staff questions regarding the current condition of the resident, receipt of the summary plan, which was agreed upon during the VTC, ease of understanding and continuing the summary plan, and any concerns regarding the treatment plan or the resident.

Specific education about the intervention and its implementation was developed for each RACF facility and ED and there was the option of more than one session available to ensure nurses who would be responsible for undertaking a VTC from the RACF/ED could access the education during a rostered day. The education sessions were flexible and were mostly facilitated person to person in RACFs. However, due to the impact of COVID-19 and the associated social distancing requirements, two education sessions were held via video conferencing and four were held at an offsite venue external to the RACFs. The implementation education encompassed a range of activities such as a structured communication framework that is used in the LHD for clinical handover (ISBAR) outlines the following; Introduction, Situation, Background, Assessment and Recommendations (ISBAR) [30]. This ensured the RACF staff provided all relevant information over the phone and that the ASET nurse recorded this structured information electronically during the call. Other aspects of the education included; simulated role-playing in VTC, on-site visits to emergency departments for RACF nurses, reciprocal visits to residential aged care facilities by ASET Nurses, and the use of instructional and informational videos. A proof of concept was undertaken during 2018 over four months implementing VTC to four RACFs, providing a structured educational plan to RACF/ASET nurses resulting in a 27% reduction in hospital transfers. This reduction served as the basis for designing and implementing the strategies outlined in the formal study discussed in this paper.

### Implementation

The PACE-IT implementation strategies were evidence based, incorporating engagement, facilitation, [31], resource development and deployment [32] and monitoring and feedback [33]. Initially, the implementation plan allowed for the onset of winter to be approximately the mid-point of the study. To allow the aggregated control and intervention time periods across steps the study needed to involve equal amounts of winter/non-wintertime thus accounting for seasonal variations during the “flu” season, therefore enabling reliability and consistency of measure. The onset of COVID-19 delayed the commencement of the PACE-IT implementation and further impacted resident presentations to ED. Fourteen months of data were analysed, from three months before the first intervention (March 2020) until three months after the last intervention (April 2021). Further details of implementation strategies are available in Supplementary File 4.

### Primary outcome

The rate of ED presentations from RACFs per 100 RACF beds during ED ACE hours

Secondary outcomes:

1. Presentations to ED within 48 h post ED ACE/VTC
2. RACF staff perceptions of VTC useability and acceptability
3. Uptake of ACE/VTC

Process measures to monitor potential adverse events are presented, using responses to a follow-up phone call from ED staff to RACF staff who attended a VTC within the previous 24 h, where the resident did not present to ED.

Data to assess the primary and secondary outcomes and process measures for LHD A were obtained from an ED Electronic Patient Management System (PMS). Data from LHD B were obtained monthly via an onsite PACE-IT liaison person. Two of the RACFs (Rural 1/2) were separate RACFs but from the same company and co-located. Data from these sites were combined in PMS as one RACF (with the same RACF ID).

Data measuring RACF staff perceptions of VTC useability in both LHDs were collected from an anonymous electronic survey (including demographic data) emailed to every RACF nurse who attended a VTC, to assess the useability and acceptability and any technical issues involved with using the VTC.

The PACE-IT Research Project Staff Survey (Supplementary File 5) modified from the Telehealth Useability Questionnaire (TUQ) [34] was piloted amongst various staff members to ensure that the survey question selection was refined before it was circulated. The TUQ was found to have “good” to “excellent” reliability with a Cronbach’s alpha coefficient of 0.81 and was demonstrated to perform well in previous studies for relevant study populations [34] adding validity to this study.

### Statistical methods

Descriptive statistics for episodes of care occurring in ED ACE hours (Monday to Friday, 8am to 4 pm) are presented as count and percentages for categorical data. Mean and standard deviation (SD) and median (with minima and maxima) for continuous data are presented. The measures presented are the proportions of episodes of care by type of contact.

This was an intention to treat analysis. Intention to treat in this context means that all calls which occurred after the implementation of the intervention were deemed to be VTC calls regardless of whether they were a phone call or VTC.

The number of ED (physical) presentations were aggregated at the RACF level for each study month. Months were classified as pre or post-implementation, based on the month that the intervention was first implemented. There was an RACF “settling in period” of two weeks that allowed close surveillance of barriers and enablers that didn’t affect the VTC count. The month that the intervention was first implemented in an RACF, and all following months, were classified as post-implementation; regardless of on which day the implementation occurred. The rate of ED presentations per 100 RACF beds was modelled using a mixed effects negative binomial regression model with the number of ED presentations per month for each RACF as the outcome and the log number of beds per 100 RACF beds as the offset. The analysis included terms for study month (as categorical) and implementation status (pre or post-implementation) as fixed effects. The categorical study month variable was used to adjust for potential effects of underlying secular trends at each discrete time point which was assumed to be common among all sites. Random intercepts for cluster and RACF nested within cluster were included in the model. The difference in rates of ED presentations between pre and post-implementation periods within ED ACE hours was examined and reported as incidence rate ratios (IRR) with 95% CI. The difference in rates of ED presentations between pre and post-implementation periods during ED ACE hours combined with non-ED ACE hours was also reported.

The secondary outcome was presentations to ED within 48 h and not the same-day as the episode of care for RACF residents who had not presented to ED after a VTC. This outcome was assessed by auditing the results of the 24-h phone call and comparing to the reason for the ED presentation to decide if an adverse event had occurred.

A disadvantage of modelling the rate of ED presentations per month per RACF is that it does not consider the actual number of episodes of care. Episodes of care where an ED ACE/VTC was conducted, and the resident did not attend ED are not explicitly included as data when modelling the rate of ED presentations. Therefore, in a post-hoc analysis, a logistic regression was conducted on all episodes of care with ED presentation (yes/no) as the outcome, variables for pre and post-implementation and study month fitted as fixed effects and adjusting for resident gender and age. This analysis defined the pre/post-implementation status based on the actual date of the implementation, not the month of implementation, and estimated the odds of an episode of care resulting in an ED presentation post-implementation relative to pre-implementation. Random intercepts for cluster and RACF nested within cluster were included in the model.

To assess uptake of VTC, the proportion of ED ACE calls conducted pre-implementation and the proportion of ED ACE/VTCs post-implementation are presented.

## Results

### Demographic characteristics

There were 333 RACF episodes of care during ED ACE hours pre-implementation and 421 RACF episodes of care during ED ACE hours post-implementation. Demographic characteristics of participants appeared to be similar between pre and post-implementation periods.

Demographic characteristics of residents this is reflected in the demographics in Table 1 with the minimum age as low as 35, the gender is not representative of the Australian RACF cohort, however participating sites encompassed rural and metropolitan areas to reflect the diversity of RACF residents. The characteristics of episodes of care are presented in Table 2 for the pre and post-implementation periods.

**Table 1:** Characteristics of residents^a^

**Characteristic**	**Response/statistic**	**Pre-implementation** *(N* = *333)*	**Post-implementation** *(N* = *421)*
Resident age	mean (SD)	83 (9)	84 (8)
	Median (min, max)	85 (34, 100)	85 (45, 102)
Resident gender	Female	171 (51%)	224 (53%)
	Male	162 (49%)	197 (47%)
Aboriginal and/or Torres Strait Islander peoples		11 (3%)	16 (4%)

^a^based on 98 months of data pre-implementation (average of 6.1 months per RACF) and 126 months of data post-implementation (average of 7.9 months per RACF)

**Table 2:** Characteristics of episodes of care^a^

**Characteristic**	**Response/statistic**	**Pre-implementation** *(N* = *333)*	**Post-implementation** *(N* = *421)*
Episodes of care	ED ACE/VTC with no ED presentation	35 (11%)	72 (17%)
	Presented to ED (after ED ACE/VTC)	63 (19%)	150 (36%)
	Presented to ED (without ED ACE/VTC)	235 (70%)	199 (47%)
ED ACE/VTC		98 (29%)	222 (53%)
Crude annual rate of ED presentation per 100 RACF beds ^a^		41 per 100 RACF beds	37 per 100 RACF beds

^a^ based on 98 months of data pre-implementation (average of 6.1 months per RACF) and 126 months of data post-implementation (average of 7.9 months per RACF)

### Uptake of ED ACE/VTC

Pre-implementation, there were 98 ED ACE calls made, 29% of all episodes of care. Thirty-five of these calls resulted in the RACF resident not presenting to the ED. Overall, 235 of the 333 RACF episodes of care (71%) resulted in an ED presentation (with or without a prior ACE call).

Post-implementation, there were 222 ED ACE/VTCs made, 53% of all episodes of care, a highly significant increase in the percentage of ED ACE calls made, with or without the addition of VTC (*p* < 0.01). Of these calls, 72 resulted in the RACF resident not presenting to the ED. Overall, 199 of the 421 RACF episodes of care (47%) resulted in an ED presentation (with or without a prior ED ACE/VTC). Descriptive statistics of ED presentations are presented in Table 1.

### Rate of RACF resident ED presentations pre/post-implementation

The results of the mixed negative binomial regression of the rate of ED presentations (per 100 RACF beds) are presented in Table 3. Post-implementation, there was a non-significant 29% decrease (IRR [95% CI]: 0.71 [0. 46, 1.10] *p* = 0.122) in the rate of ED presentations (per 100 RACF beds) during ED ACE hours compared to pre-implementation.

**Table 3:** Rate of ED presentations pre and post-implementation

	**Negative binomial regressions **^**a**^ **Adjusted estimates **^**b**^	
	**IRR (95% CI) **^**c**^	***p*-value**
ED presentation during ED ACE hours	0.71 (0.46, 1.1)	0.122
All ED presentations (ED ACE + non-ED ACE hours)	0.96 (0.65, 1.41)	0.844
	**Logistic regression **^**d**^ **Adjusted estimates **^**e**^	
	**OR (95% CI) **^**f**^	***p*-value**
ED presentation during ED ACE hours	0.31 [0.11, 0.87]	0.025

^a^Negative binomial regressions are based on episodes of care resulting in an ED presentation (*n* = 333 pre-implementation, *n* = 421 post-implementation)

^b^Estimates from negative binomial regressions, adjusted for study month and implementation status

^c^Rate per 100 RACF beds

^d^Logistic regressions are based on all episodes of care within ED ACE hours, whether it resulted in an ED presentation or not (*n* = 333 pre-implementation, n = 421 post-implementation)

^e^Estimates from a logistic regression, adjusted for study month and implementation status

^f^Odds ratio of an episode of care resulting in an ED presentation post relative to pre implementation

When ED ACE and non-ED ACE hours combined were analysed, there was a non-significant 4% decrease post-implementation, (IRR [95% CI]: 0.96 [0. 65, 1.42], *p* = 0.844) in the rate of ED presentations (per 100 RACF beds) compared to pre-implementation.

### Post-hoc analysis of episodes of care resulting in an ED presentation

The results of the logistic regression analysis of episodes of care which resulted in an ED presentation or not, showed a significant post-implementation reduction of 69% in the odds of an episode of care resulting in an ED presentation (OR [95% CI]: 0.31 [0.11, 0.87], *p* = 0.025). Results are shown in.

### Presentations to ED within 48 h post VTC to identify adverse events

Pre-implementation, there was one occurrence where an ACE call was made, and the agreed plan was for the resident to remain and be monitored by staff in the RACF. However, the resident had an unplanned presentation to the ED within 48 h of the initial call. Post-implementation, there were two occurrences where a VTC was made, and the agreed plan was for the resident to remain and be monitored by staff in the RACF and the resident had an unplanned presentation to the ED within 48 h of the initial VTC. For the post-implementation residents, there were no concerns raised about either resident’s condition by RACF staff during the 24-h follow-up phone calls. Comments received re the residents’ condition during the 24-h follow-up phone call were “Neuro observations stable” and “will follow up with ACE if any issues”. Of the two residents that presented to ED within 48 h neither had any reported adverse events; one resident required referral for wound care follow-up and the other required investigation for hip pain that was not initially reported in the VTC. No analysis was conducted to compare within 48 h presentations pre and post-implementation as there were insufficient numbers to justify this.

### RACF staff perceptions of useability and acceptability of VTC within 48 h of participating in VTC: PACE-IT Research Project Staff Survey results

There were 113 VTCs attended for the duration of the project. On each occasion when a VTC was completed, an electronic hyperlink to the PACE-IT Research Project Staff Survey was emailed to the nominated RACF contact person to disseminate to the relevant RACF staff participant for response. There were 44 responses (39%) to the PACE-IT Research Project Staff Survey (39 complete and 5 partially complete). The characteristics of the survey respondents are described in Table 4.

**Table 4:** Characteristics of PACE-IT Research Project Staff Survey respondent results

Characteristic		Respondents (*N* = 44, 39%)
Age group	Less than 30 years	12 (27%)
	31–41 years	17 (39%)
	41–50 years	11 (25%)
	51–60 years	4 (9%)
Gender	Female	34 (77%)
	Male	10 (23%)
Registered nurse Other level of staff		32 (73%) 12 (27%)
Spoke a language other than English at home Only spoke English at home		17 (39%) 27 (61%)

Of the respondents 27 (61%) had attended the face-to-face training required for the PACE-IT intervention (Sunner et al., 2020, pp. 5–6). Twenty responders (45%) considered that it took between 10- 15 min to undertake the call. Responses to the useability and acceptability of VTC in the RACF indicated that 38 responders (86%) strongly agreed or agreed that it was easy to set up and 39 (88%) strongly agreed or agreed that it was easy to use. Thirty eight responders (86%) strongly agreed or agreed that the quality of the call was acceptable. When examining the time, it took to do the call, 31 (70%) strongly disagreed or disagreed that it was too time consuming. One hundred percent of respondents indicated that a Visual Telehealth ACE call enhanced communication and 97% felt it provided a person-centred approach (one respondent was neutral so no respondents disagreed with this question). Ninety two percent of respondents strongly agreed or agreed that they were satisfied with the agreed resident management plan (the remaining 8% were neutral about the management plan).

Responses to the PACE-IT survey indicate that some participants found the initiation of calls challenging, sometimes due to network issues. Most appreciated the person-centred approach of the service in relieving anxiety among residents. Visual telehealth calls were considered useful in avoiding unnecessary transfers, and some respondents suggested assigning dedicated devices for these calls. The service was seen as a positive step toward a more person-centred care approach and praised for providing better decision-making support, despite occasional audio and video connectivity issues. Participants reported an overall positive experience with the PACE-IT MoC and expressed a desire for weekend service availability and to expanding the service to more departments, such as mental health and dental clinics. Additionally, the MOC was rated as improving communication between the resident, RACF and ASET nurse.

### Follow-up phone calls to RACF staff 24 h after a VTC with no ED presentation

Forty VTCs were conducted where the RACF resident did not attend the ED. Follow-up phone calls were conducted with RACF staff 24 h after a VTC for residents who were not transferred to ED. This was an additional quality measure, along with the 48 h post VTC ED presentation data check. RACF staff were asked questions about the health/well-being of the resident. Thirty-four (83%) of the RACF responders were registered nurses. The average age of the resident was 86 with the age range being 62 to 99 years. Table 5 describes the follow up questions and responses. Of the 40 who responded to the questions, 37 (92%) felt the resident’s condition had remained stable or had improved. For question 2, 20 responses (51%) were positive indicating a consultation summary plan had been received; for questions 3 to 6, over 95% of responses to the management plan or intervention were rated as either beneficial or successful in achieving its intended goals or outcomes for the resident.

**Table 5:** Responses to 24-h follow-up phone calls to RACF staff following a VTC

	**Follow-up questions**	**N**	**Deteriorated**	**Improved**	**Stable**
1	How has the resident been feeling since our visual telehealth consultation yesterday?	40	8%	37%	55%
		**N**	**No response**	**Yes**	**No**
2	Did you receive the Visual Telehealth Consultation summary plan?^a^	39	1	51%	49%
3	Were you able to follow the Visual Telehealth Consultation plan?^a^	37	3	95%	5%
4	Did the Visual Telehealth Consultation plan address all the issues for the resident?^a^	37	3	97%	3%
5	Do you have any concerns about the treatment plan for the resident?^a^	38	2	3%	97%
6	Are you happy to continue on the current plan for the resident?^a^	39	1	95%	5%

^a^Not all questions were answered by all participants, so the denominator varies

## Discussion

The PACE-IT project analysis demonstrated that, with the addition of VTC to the usual MOC, there was an overall 29% relative reduction of the rate of ED presentations (per 100 RACF beds) when an ED ACE/VTC call was made within ED ACE hours (when this service is accessible). This result was not statistically significant; however, it represents a clinically important decrease in exposure of RACF residents to the ED environment. All episodes of care from RACFs were supposed to have an ACE call to the ED prior to attending the ED. Pre-implementation, 333 episodes of care occurred in ED ACE hours, only 98 of these had a prior ACE call made to the ED (29% of episodes of care). Post-implementation, 421 episodes of care occurred in ED ACE hours, and 222 of these had an ED ACE/VTC (53% of episodes occurring within ED ACE hours), reflecting a highly significant increase in the use of the ED ACE service, with or without VTC.

The study aimed to detect a 35% reduction in ED presentations per 100 RACF beds from a baseline rate of 82 ED presentations per 100 beds, which was both clinically meaningful and practical. The observed 29% reduction, while not statistically significant, may be due to limited statistical power caused by a slightly smaller true effect and the lower baseline rate of ED presentations (around 41 per 100 RACF beds annually). The PACE-IT project started in 2019 during the COVID-19 pandemic, which led to fewer ED visits overall.

This 29% reduction in ED presentations highlights the benefits of dedicated, constant support and education through visual assessments. Notably, the most significant reduction in hospital transfers (29%) occurred when ASET nurses conducted VTCs during ED ACE hours, in contrast to a 4% reduction during non-ED ACE and ED ACE hours combined. These findings suggest the potential to expand ASET nurse/ED ACE hours for more comprehensive coverage throughout the day and evening. Findings demonstrate that when RACF nurses undertake ED ACE/VTCs there is a reduction in RACF resident ED visits.

Successful implementation relies on RACF staff's willingness and motivation to utilise the ACE service and the visual component of the consultation. VTCs helped develop relationships between RACF and ED nurses [35] and these synergies further act to normalise ED ACE/VTC and facilitate its adoption as usual care. This is evidenced through the survey results where 100% of respondents indicated that the VTC service enhanced communication and 97% felt it provided a person-centred approach. Further motivation for RACF staff to utilise VTC is the fact that the resident has access to an additional benefit of advanced levels of care that support and strengthen care decision-making processes including the perceived access to specialised care using VTC [35]. When dealing with complex or acute health issues that exceed the scope of routine care, RACF nurses may see the value in involving ACE/VTC to consult with experienced clinicians, including Emergency Department doctors. This access to expert guidance can empower nurses and enhance the quality of care provided to residents.

Post-implementation the top five reasons for VTC calls for ED presentations from RACF residents were for; abdominal pain, chest pain, acute upper respiratory infection, low back pain, urinary tract infection. However not all ACE calls were deemed appropriate for VTC. There were times where VTC was deemed inappropriate, for example, if the resident had; an exacerbation of behavioural and psychological symptoms of dementia with excessive aggression that would be further agitated by a VTC, evidence of a haemorrhage, excessive pain, unexpected loss of consciousness/ sense of urgency, actively fitting/ seizure or simply a follow up call (e.g. medication script, x-ray report etc.). For this reason, it was important to allow for staff discretion and flexibility in choosing whether to use VTC during the ACE call and this needs to be considered by staff in both the ED and the RACF. Based on post-implementation feedback exclusion criteria have been developed and added to VTC guidelines.

There were many missed VTC opportunities for which the RACF nurses presented a range of responses including: the iPad was not charged or no compatible device was available for a VTC, there was an agency nurse on duty who was new and not familiar with the procedure, the RACF nurse refused to attend a VTC as they were not trained, there was a technical system failure, the resident was already in an ambulance and on their way to hospital and the staff were “just handing over”.

Including a VTC option as part of an assessment service for RACF residents that enhances telephone outreach services is a practical strategy. Nurse-led outreach models that include partnerships between ED and RACFs using existing ED Aged Care Specialist services, together with protocol driven guidelines have been shown to be both feasible [18, 36, 37] and cost effective [19]. Our project was implemented with existing stable Wi-Fi in all facilities and inexpensive accessible tablet-based technology. The low numbers of residents with unplanned presentations to the ED within 48 h of a VTC (where a same-day ED presentation was not made) suggests this MOC provides a safe high quality care options in the management of residents.

The PACE-IT intervention has the potential to improve the clinical care and quality of life of the frail older person and reduce ED presentations and their associated risks. It has provided evidence that can be used to inform sustainable change and translation into practice. PACE-IT has demonstrated how the change has been achieved and highlighted success factors for scalability and sustainability. Specifically, it has identified how PACE-IT reduces ED presentations and admissions to hospital for residents of RACFs [38]. It will inform the review of processes and the development of Policy and Guidelines that will integrate PACE-IT into existing service models.

These findings have direct clinical implications as well as policy implications for those setting standards for the Australian National Safety and Quality Health Service Standards [39]. In particular, Standard 5, “Comprehensive Care” for RACF residents, ensuring clinically appropriate care at the right time in the right place that meets individual’s needs and wishes, and reduces risk of harm. Alongside this is Standard 2 “Partnering with Consumers” [39] where visual telehealth links positive patient experiences, high-quality health care and improved health care safety. The continuation of the service and how policy can be modified to support ongoing implementation will be the ongoing concern.

This study has provided evidence for translation by generating new knowledge about how PACE-IT, provided to RACF residents with a general range of acute conditions, reduces ED presentations. The PACE-IT intervention can be transferred to many settings within metropolitan, rural and remote areas and in differently sized EDs and RACFs. However, the challenge is to ensure current interventions are embedded as normal practice to ensure that similar MOC are sustained into the future. Challenges include the lack of ongoing funding to scale up the intervention, incentives for implementation of leadership in this area and availability of appropriate champions to influence successful implementation and outcomes, common issues found in other studies [16, 40]. Furthermore, the competing workloads, lack of training for RACF nurses, depleting aged care workforce [8] and insufficient medical back up and support can also hamper the translatability into routine care.

PACE-IT establishes a governance structure that engages all stakeholders (including consumer representation), a structure that is easily replicable nationally. Current members of the PACE-IT governance group have enthusiastically embraced their role in developing the study design and PACE-IT model and in making themselves available for consultation, indicating a high level of engagement and ongoing commitment. Establishment of the VTC upskilling education program and additional resources (e.g., Guidelines, RACF unit-posters, resident/family information brochures, media-briefing) will facilitate local engagement ensuring easy transfer and adaptability of PACE-IT across sites/LHDs. Integral to the success of this MOC is dedicated leadership that can drive the intervention and have time to review the processes and data whilst continuing to educate and liaise with ASET and RACF nurses.

Once embedded, EDs across NSW may consider expanding to a 24/7 ACE service which would require additional resourcing. The cost/consequence analysis in this study (to be reported separately) will inform the feasibility of this action.

This study represents a significant contribution to the literature by both presenting its findings and outlining a novel approach in providing care for RACF residents. The adoption of VTC has demonstrated a noteworthy reduction in hospital admissions for residents of residential aged care facilities, underscoring its clinical significance. These findings strongly support the broader implementation of visual telehealth consultations. This justification emphasises the importance of integrating VTC as a vital tool to aid RACF clinicians in their decision-making processes.

### Strengths and limitations

Different methods of data collection were utilised for this study to ensure rigour. Primary outcome data measuring RACF ED presentations and VTCs were collected monthly from PMS and included information on demographic characteristics of RACF residents, presenting problem and call outcome (ED presentation or alternative care pathway). The generalisability of this study was ensured by engaging with different locations and contexts, and by selecting EDs for their metropolitan and rural locations.

At any given time, the randomised mixture of sites ensured that some sites had the intervention, and some did not, thus accounting for secular variations. The fixed effect for step will control for a common underlying secular trend across all clusters [27].

The study was unlikely to have achieved the planned statistical power, due to the lower overall rate of ED presentations because of the impact of the COVID pandemic and subsequent lockdowns, as well as a slightly lower than anticipated intervention effect. However, the overall reduction in ED presentations per 100 RACF beds of 29%, while not statistically significant, demonstrates an important clinical effect.

## Conclusion

Despite the implementation of the study becoming hampered by the onset of COVID-19, study findings have shown feasibility and clinically significant reductions in RACF resident ED presentations when VTC is included in communications between RACFs and EDs. This clinical significance cannot be understated. Any reduction of resident presentations to a busy ED is of huge benefit to healthcare overall, but more so to the individual older person who can recover safely in their own RACF.

### Supplementary Information

**Additional file 1.**

**Additional file 2.**

**Additional file 3.**

**Additional file 4.**

**Additional file 5.**

## Data Availability

The datasets used during the current study are available from the Australian Data Archives at the Centre for Social Research and Methods at the Australian National University. Repository website: https://ADA.edu.au.

## Abbreviations

ACE: Aged Care Emergency telephone only calls from RACF for consultation, provided 24 h a day, by the ASET nurse in the ED from 8-4 pm and after hours by a separate health care provider
ED ACE: Within ASET rostered hours: Monday to Friday, 8 am to 4 pm
non-ED ACE: A separate health care provider for after-hours ACE calls
VTC: Visual Telehealth Consultation is an ED ACE call with the addition of a video conferencing platform for interactive assessment and clinical decision making
ED ACE/VTC: An ED ACE telephone call with or without a VTC
ED: Emergency Department
ED presentation: An ED presentation was defined as an RACF resident transfer to ED (“in-person” presentation to the ED)
Episode of care: An episode of care includes all instances where an ED medical record was created for a RACF resident when the resident was transferred to the ED, with or without an ED ACE call/VTC, or when an ED ACE/VTC was attended with no resident transfer to the ED
GP: General Practitioner
IRR: incidence rate ratio
OR: Odds ratio
LHD: Local Health District
MOH: Ministry of Health
PACE-IT: Partnerships in Aged Care Emergency using Interactive Telehealth
PMS: Patient Management System
RACF: Residential Aged Care Facility
TUQ: Telehealth Usability Questionnaire
48: hour presentation an “in-person” presentation to the ED within 48 h, but not on the same day, following a VTC where the resident did not attend the ED
Unnecessary ED Presentation: An unnecessary Emergency Department (ED) presentation refers to a situation in which an individual seeks care or treatment at an ED for a medical condition or issue that could have been adequately addressed and managed in the a less acute setting e.g. the RACF
