# Supplementary material for: Implementation and evaluation of a nurse-led intervention to augment an existing residential aged care facility outreach service with a visual telehealth consultation: stepped-wedge cluster randomised controlled trial
Source: BMC Health Serv Res. 2023 Dec 18;23:1429. doi: 10.1186/s12913-023-10384-z (PMC10726593; doi:10.1186/s12913-023-10384-z)
Supplement: Supplementary file 1 — Additional file 1. [file 12913_2023_10384_MOESM1_ESM.docx]

**Supplementary File 1**

**The Essential Elements of ACE (Hullick et al., 2021, p. 203) with The Addition of VTC**

| **Elements** | **ACE** | **VTC** |
| --- | --- | --- |
| A 24 hour nurse-led telephone consultation service for staff in RACFs provided by RNs in the ED Monday to Friday 8am-4pm and after hours by RNs from the local general practice organization. | 🗸 | 🗸 |
| Evidence-based algorithms for common acute symptoms and problems experienced by residents from RACFs, developed in consultation with multidisciplinary hospital and community-based providers along with RACF clinical leaders and the ambulance service. | 🗸 | 🗸 |
| If transfer is required, the telephone call also clarifies the reason for transfer to hospital through establishing the resident’s goals of ED care. | 🗸 | 🗸 |
| Using the Identity, Situation, Background, Assessment, and Recommendation (ISBAR) protocol (Supplementary File 4). | 🗸 | 🗸 |
| Once in the ED, the resident receives proactive case management under the guidance of specialist aged care nurses. | 🗸 | 🗸 |
| Empowerment of RACF staff occurs through education in communication techniques including effective clinical handover, recognition of the deteriorating patient, and the evidence-based ACE algorithms. | 🗸 | 🗸 |
| The community of practice supports relationships and collaboration across RACFs, GPs, ambulance, local hospitals, and EDs with a shared understanding of the capability of each service.  Quarterly meetings are held to identify barriers and facilitators of care.  Regular governance and operational meetings with providers and managers.  Every RACF is assigned a home ED (based on geographical location). | 🗸 | 🗸 |
| Ongoing change management and coordination for the ACE program key stakeholders. | 🗸 | 🗸 |
| **The Addition of VTC** | | |
| Visual Telehealth Consultation (VTC) to enable interactive visual and auditory consultation between RACF staff, ED ACE nurses and the resident and, family (if available) |  | 🗸 |
| Visual Telehealth consultation PACE-IT educational session plan and resources (Please refer to Supplementary File 2 & 3) which aimed to develop the knowledge, skills and confidence with the PACE-IT intervention and to develop a shared understanding of the PACE-IT process, goals and outcomes.  Educational Objectives for ED and RACF nurses were to; Develop a working knowledge of the PACE-IT process; Be aware of the role of all stakeholders; Confidently use the Visual Telehealth consultation (VTC) technology; Collaborate and communicate effectively using ISBAR via VTC; Make decisions confidently with the resident; Feel supported with the PACE-IT model of care; Problem solve when issues arise. |  | 🗸 |
| Automated ACE consultation summary letter to the GP and the RACF with a description of the ACE or VTC call | 🗸 | 🗸 |
| 24 hour follow up phone call post VTC |  | 🗸 |

Abbreviations: ACE, Aged Care Emergency; ED, emergency department; GP, general practitioner; RACF, residential aged care facility; RN, registered nurse; VTC, visual telehealth consultation; PACE-IT, partnerships aged care emergency using Interactive Telehealth; ISBAR (Supplementary File 2).
