# Supplementary figures and images for: Implementation and evaluation of a nurse-led intervention to augment an existing residential aged care facility outreach service with a visual telehealth consultation: stepped-wedge cluster randomised controlled trial
Source: BMC Health Serv Res. 2023 Dec 18;23:1429. doi: 10.1186/s12913-023-10384-z (PMC10726593; doi:10.1186/s12913-023-10384-z)

Supplementary File 2 ISBAR (Hunter New England Local Health District, 2019)


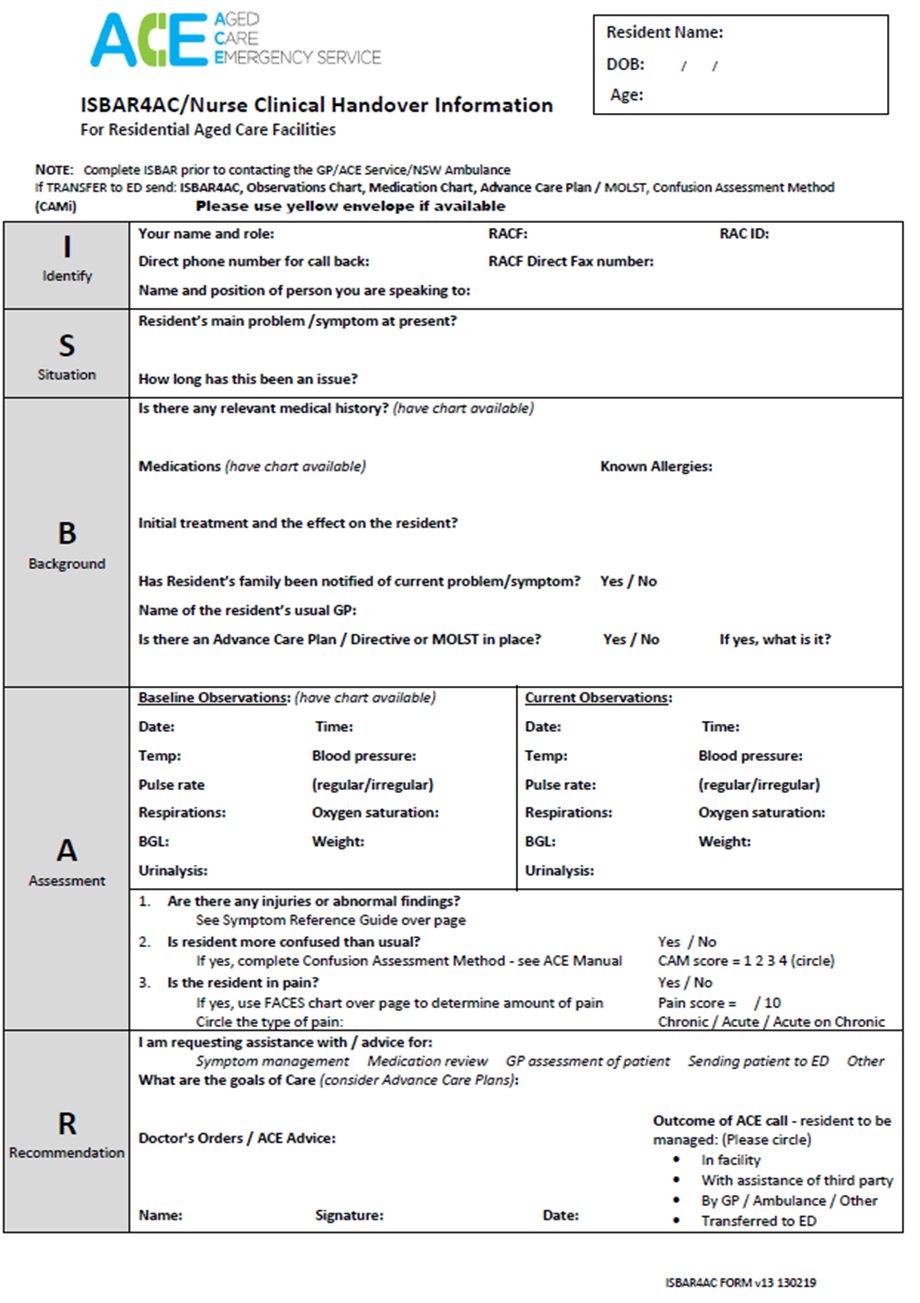

Supplement: Supplementary file 2 — Additional file 2. [file 12913_2023_10384_MOESM2_ESM.docx]

Supplementary File 3: ACE and PACE-IT MOC flowchart

ACE Flowchart


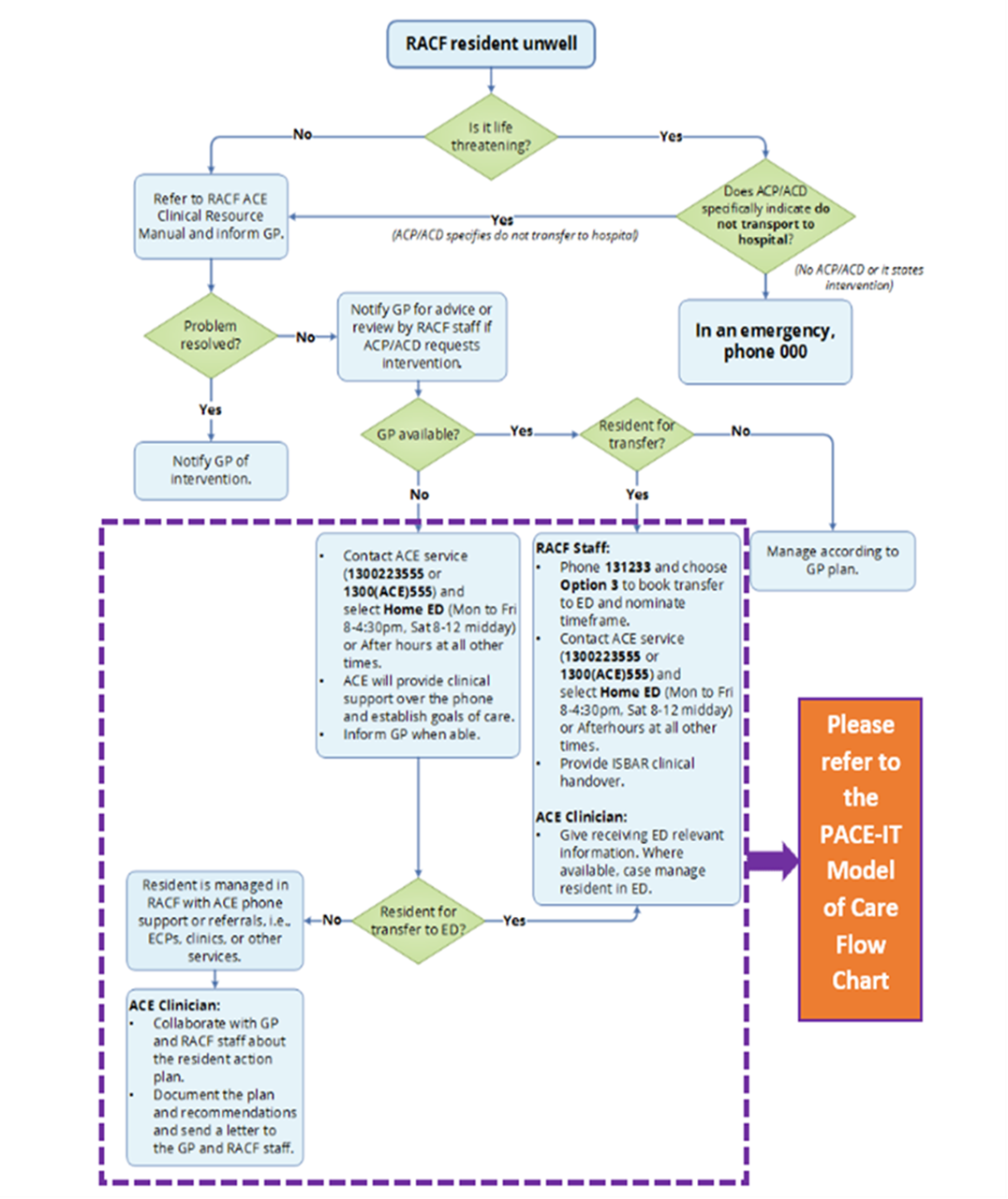


PACE-IT Model of Care Flow Chart


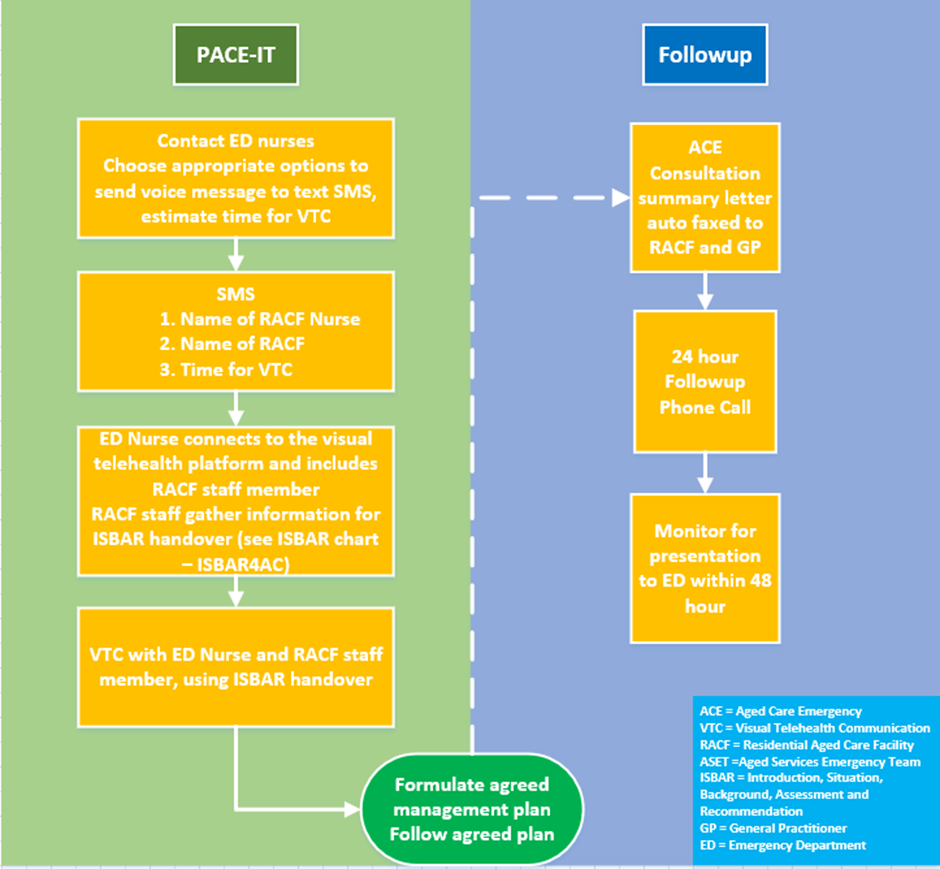

Supplement: Supplementary file 3 — Additional file 3. [file 12913_2023_10384_MOESM3_ESM.docx]

Supplementary File 5: PACE-IT Research Project Staff Survey


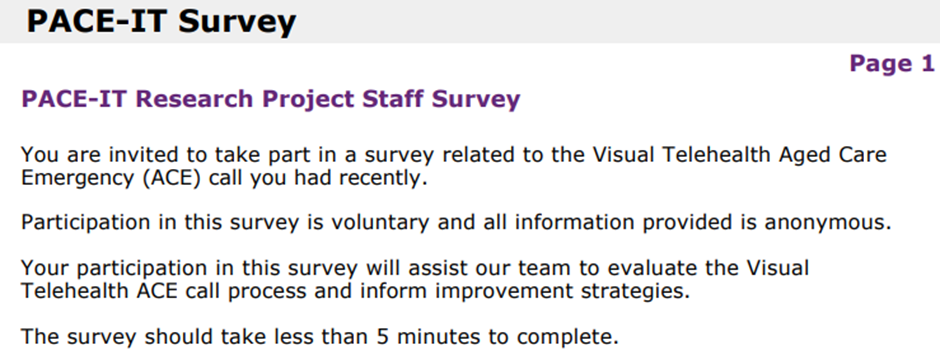


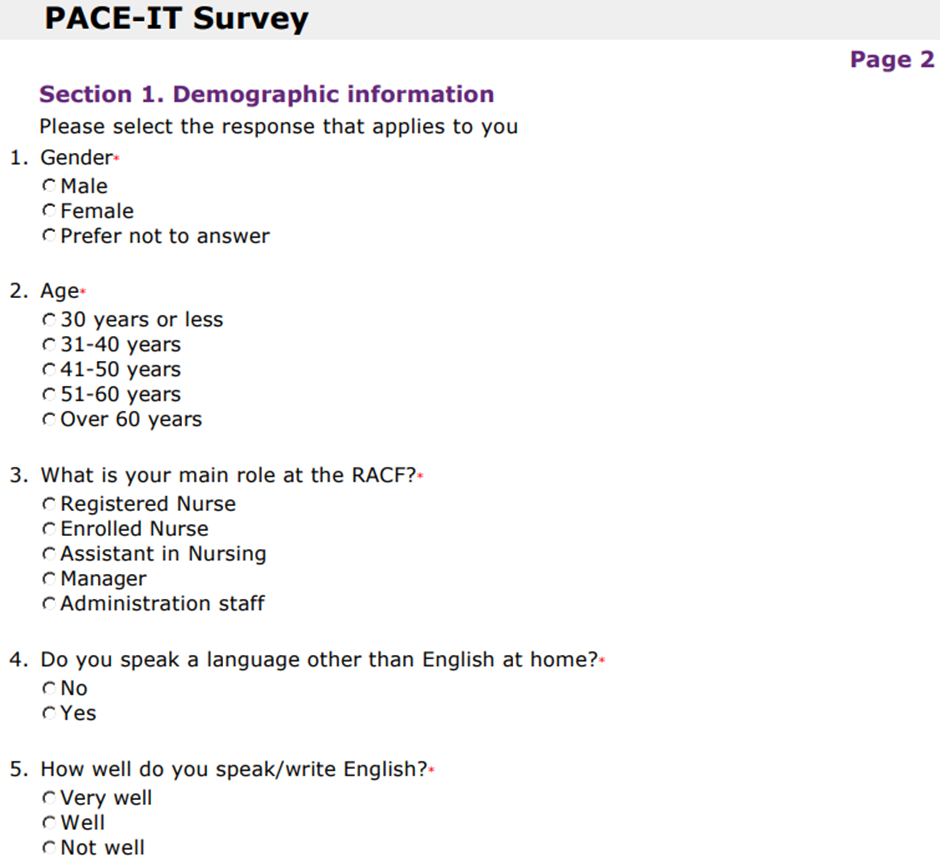


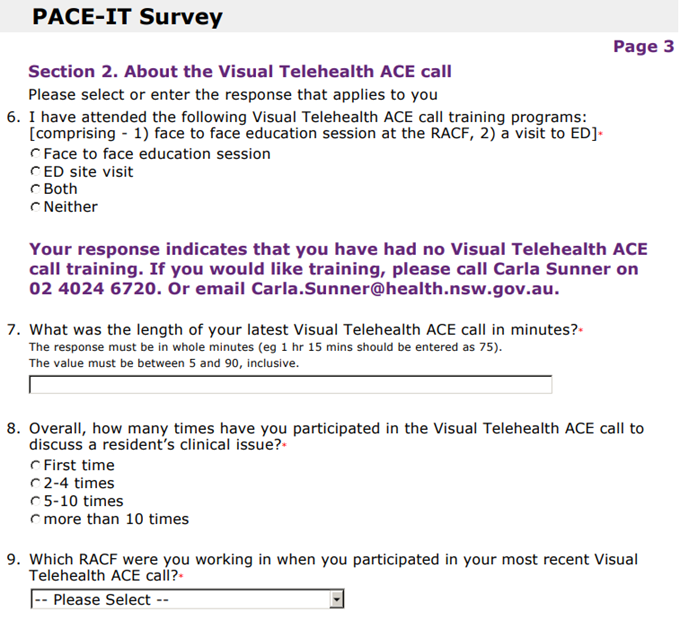


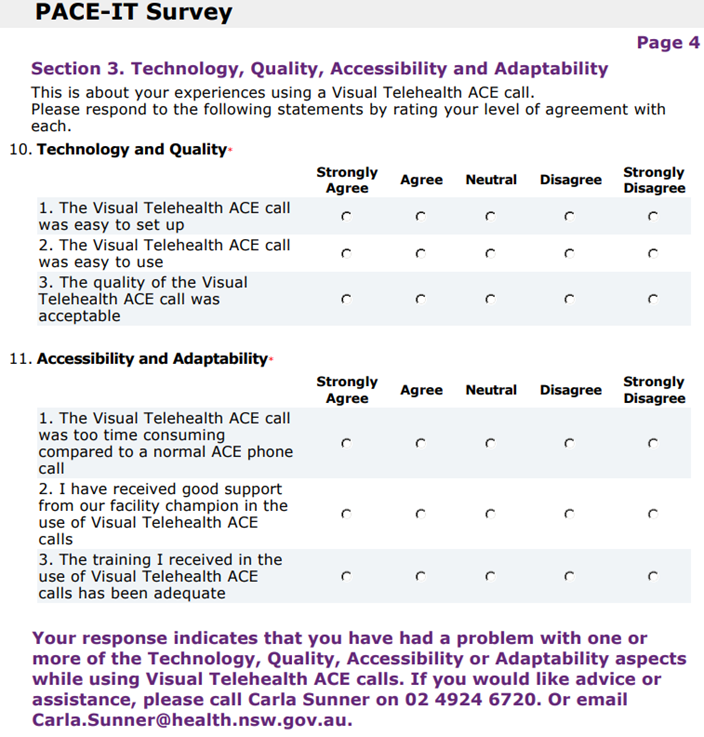


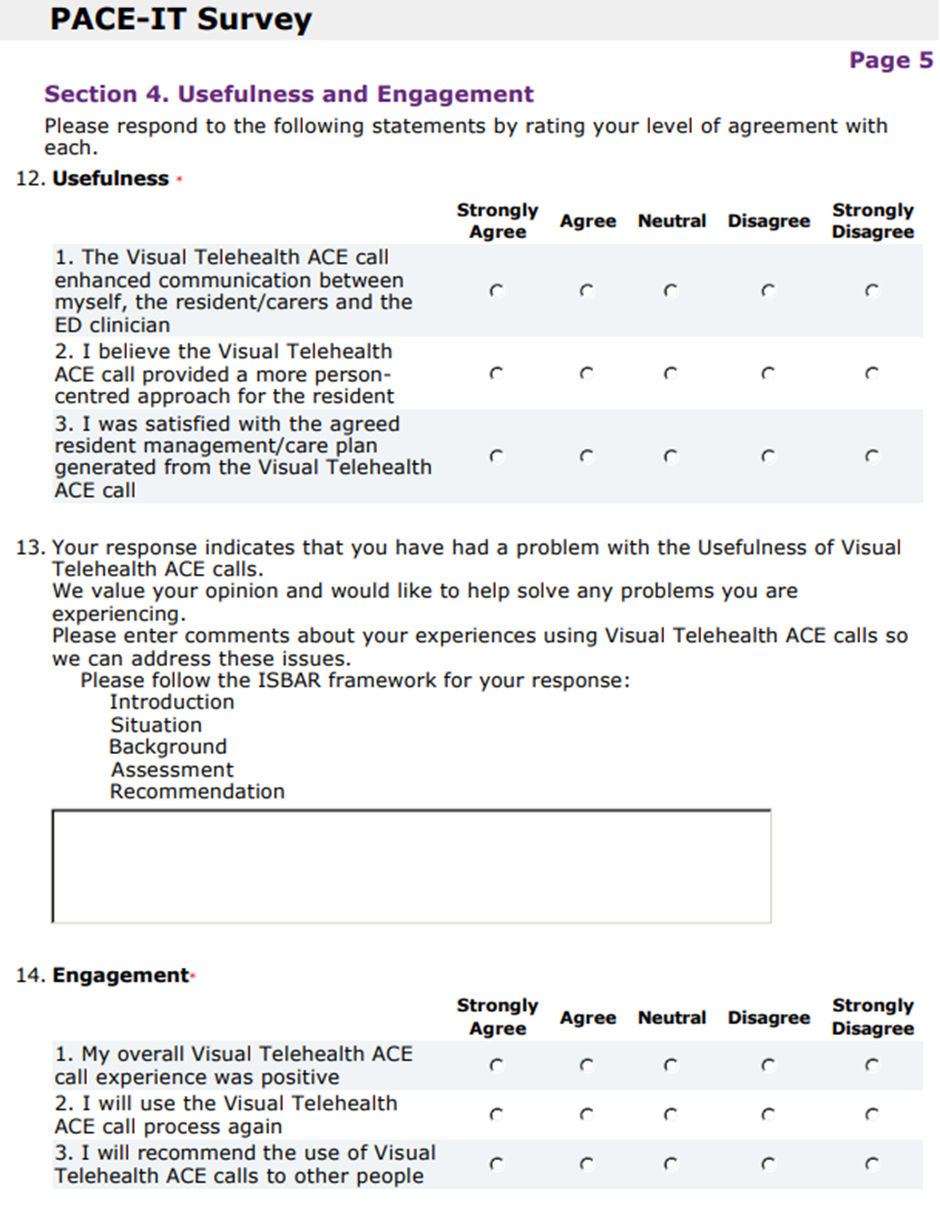


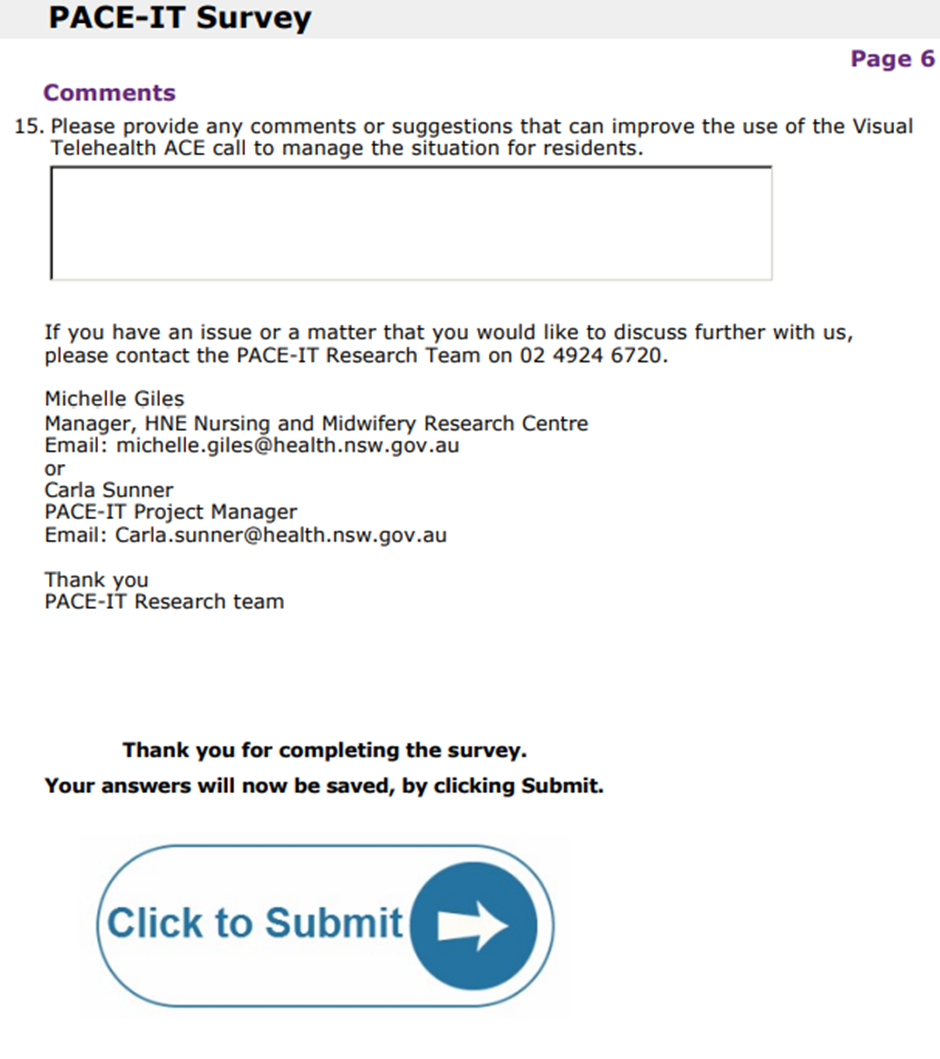

Supplement: Supplementary file 5 — Additional file 5. [file 12913_2023_10384_MOESM5_ESM.docx]
