# Supplementary material for: Implementation and evaluation of a nurse-led intervention to augment an existing residential aged care facility outreach service with a visual telehealth consultation: stepped-wedge cluster randomised controlled trial
Source: BMC Health Serv Res. 2023 Dec 18;23:1429. doi: 10.1186/s12913-023-10384-z (PMC10726593; doi:10.1186/s12913-023-10384-z)
Supplement: Supplementary file 4 — Additional file 4. [file 12913_2023_10384_MOESM4_ESM.docx]

Supplementary File 4: Intervention Implementation strategies

| Strategy | | Rationale | Who | When |
| --- | --- | --- | --- | --- |
| **Engagement** | Establish implementation groups | Improve engagement, collaboration and understanding. Identify barriers and develop context specific implementation strategies | ED and RACF nurses | Established 3 months before implementation and met monthly before and during planning/intervention/ monthly implementation and 1 month after last implementation took place |
|  | Stakeholder and Steering committee | Unlock barriers to the progress of the project. Take action to address threats to the project | LHD and MOH government executive level | 3 monthly |
|  | Information and technology | Develop user friendly systems for nurses to use, and data collection compliance. | Research team and participants | Fortnightly till reporting systems established |
|  | Research team | Continue to connect and feedback to the team, provide case studies, and discuss barriers and discuss solutions to issues | Research team | Monthly meetings |
|  | Newsletters | To continue to keep all participants and stakeholders engaged and informed of project proceedings. Continue to foster support for the project | Electronic /emailed  Mailed to General Practitioners | 3 monthly |
| **Education and resources** | Education sessions on VTC and ISBAR handover model (see Supplementary File 2) | Increase RACF staff awareness of intervention, strengthen the role of champions in progressing VTC in their workplace | RACF nurses | Initial implementation, ongoing with change of RACF staff |
|  | Nurse training about video conferencing | Familiarise ED and RACF staff with video conference equipment used in intervention | RACF staff and ED nurses at each ED | Initial implementation |
|  | ED nurses to attend RACF education days | Understand RACF context to enable implementation | ED nurses to attend RACF education days | Understand RACF context to enable implementation |
|  | RACF Aged-Care Emergency Clinical Resource Manual | Familiarise ED and RACF staff with video conference equipment used in intervention ACE/PACE-IT flow chart model (see Supplementary File 3) | RACF nurses and ED nurses at each ED | Initial implementation, RACF champions were to continue to update the available resources and encourage new staff to familiarise themselves with it |
| **Resources** | Project information brochures poster and videos | Guide ACE nurses in decision making for care of RACF residents | ED nurses | Project start and ongoing |
|  | Manual for VTC and handover model including video conferencing | Guide ACE nurses and RACF staff to normalise the VTC and handover via video- conferencing | ED nurses and RACFs | Project start |
|  | Establish video conferencing platform appropriate for use | Familiarise ED and RACF staff with video conference equipment used in intervention | ED nurses and RACFs  Each site | Project start  Realtime feedback and at implementation meetings monthly and newsletters |
| **Compliance audits and feedback** | PACE-IT Research Project Staff Survey (see Supplementary File 5) results | Address technology issues as soon as possible to ensure better integration | Nurses that participate in a VTC | Feedback via RACF champions and phone/email contact |
|  | Implementation meetings | Improve operations and useability and acceptability of VTC. Round table discussion concerning issues, feedback and case studies via videoconferencing | Champions from all sites to attend | Monthly for the duration of the project |
|  | Daily reports | Proactive support for participants by monitoring daily compliance and empower staff to continue with implementation strategies. | Research team to participants | Feedback via RACF champions and phone/email contact |
